# Supplementary material for: Facile Controlled Synthesis of Pd-ZnO Nanostructures for Nitrite Detection
Source: Molecules. 2022 Dec 23;28(1):99. doi: 10.3390/molecules28010099 (PMC9822311; doi:10.3390/molecules28010099)
Supplement: Supplementary file 1 [file molecules-28-00099-s001.zip › molecules-2090500-supplementary.pdf]

Supporting Information

Facile controlled synthesis of Pd-ZnO nanostructures for nitrite detection

Yaojuan Hu *, Fengyun He, Changyun Chen, Changli Zhang and Jingliang Liu *

School of Environmental Science, Nanjing Xiaozhuang University, Nanjing 211171, China

* Correspondence: hyj_sxnu@126.com (Y.H.); jingliangliu@njxzc.edu.cn (J.L.)

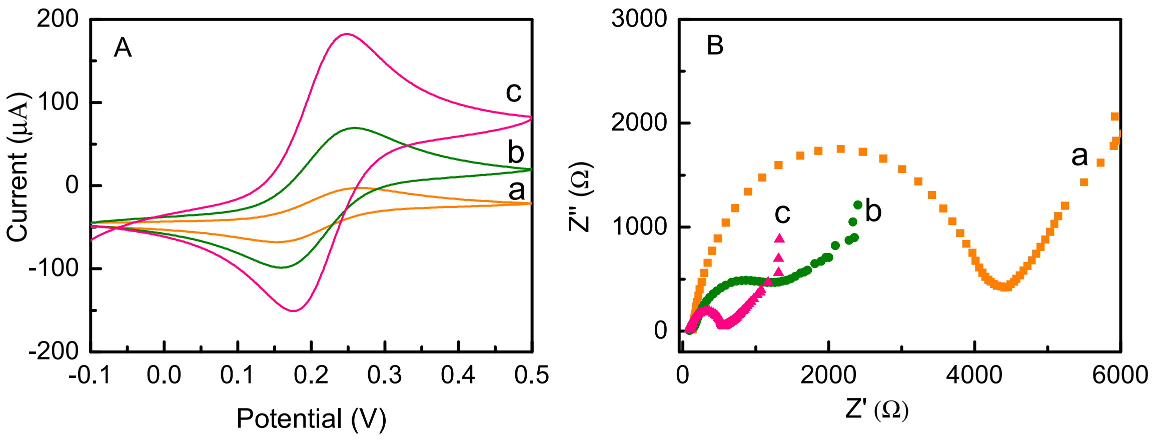

Figure. S1 Cyclic voltammetry curves (A) and Niquist plots of EIS (B) of ZnO (curve a), Pd (curve b), and Pd-ZnO (curve c) modified electrode in 0.1 M KCl solution containing 5 mM $Fe(CN)_6^{3-/4-}$ redox probe.

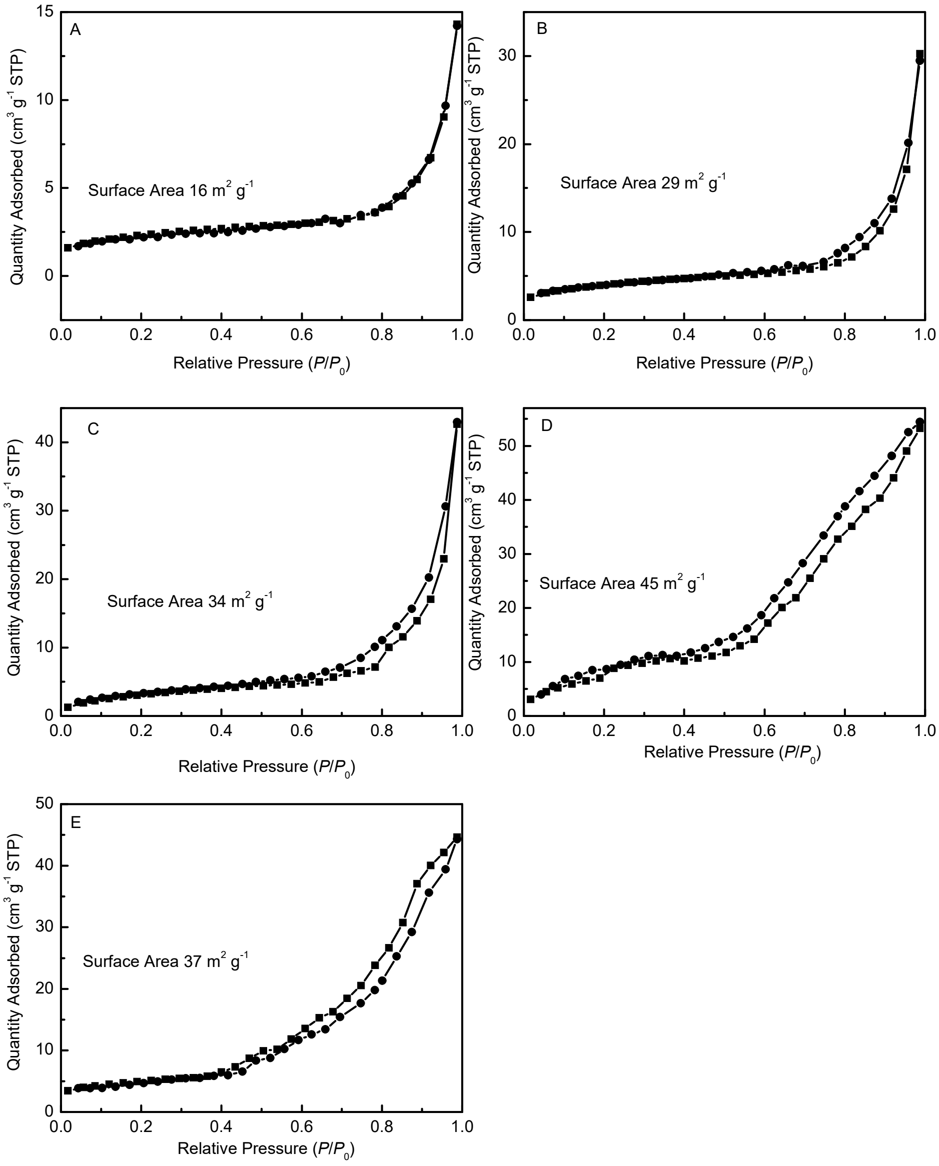

Figure. S2 Nitrogen adsorption-desorption isotherm plots of different Pd-ZnO nanostructures fabricated at different pH. The pH of the reaction solution was about 6 (curve a), 7 (curve b), 8 (curve c), 9 (curve d) and 10 (curve e) , respectively.

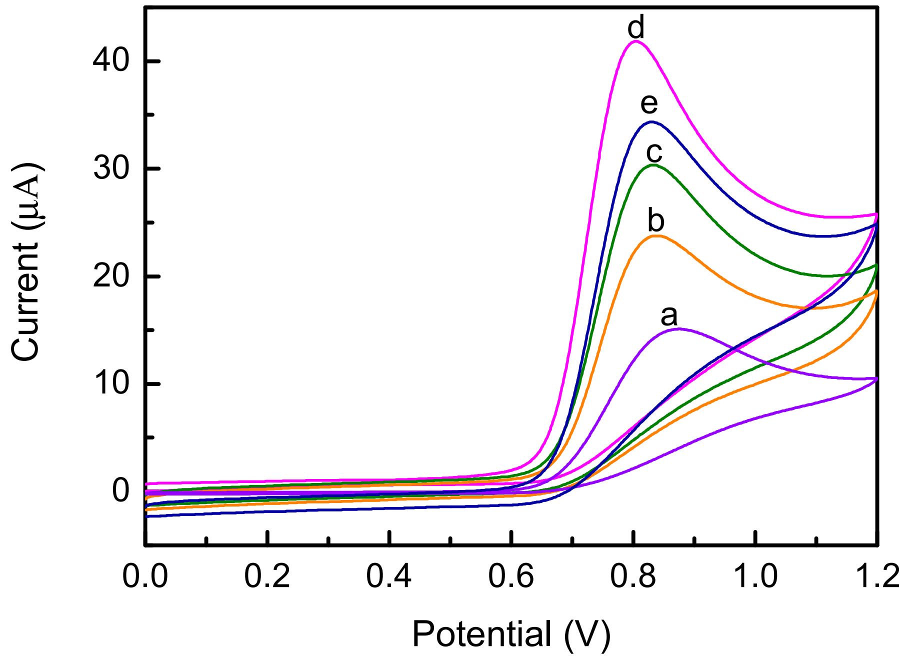

Figure. S3 Cyclic voltammograms of different Pd-ZnO nanostructures fabricated at different pH in PBS containing 0.5 mM nitrite. The pH of the reaction solution was about 6 (curve a), 7 (curve b), 8 (curve c), 9 (curve d) and 10 (curve e) , respectively. The scan rate is 50 mV/s.

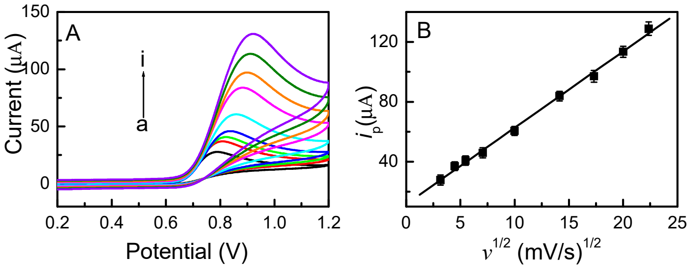

Figure. S4 (A) Cyclic voltammograms of Pd-ZnO/GCE in PBS (pH 7.0) containing 0.5 mM nitrite at different scan rates: the scan rate is 10 (a), 20 (b), 30 (c), 50 (d), 100 (e), 200 (f), 300 (g), 400 (h), 500 mV/s (i), respectively. (B) The linear relationship between the oxidation peak current (i_P) and the square root of scan rate ($v^{1/2}$). (C) The linear relationship between the oxidation peak potential (E_P) and the natural logarithm of scan rate (ln v).

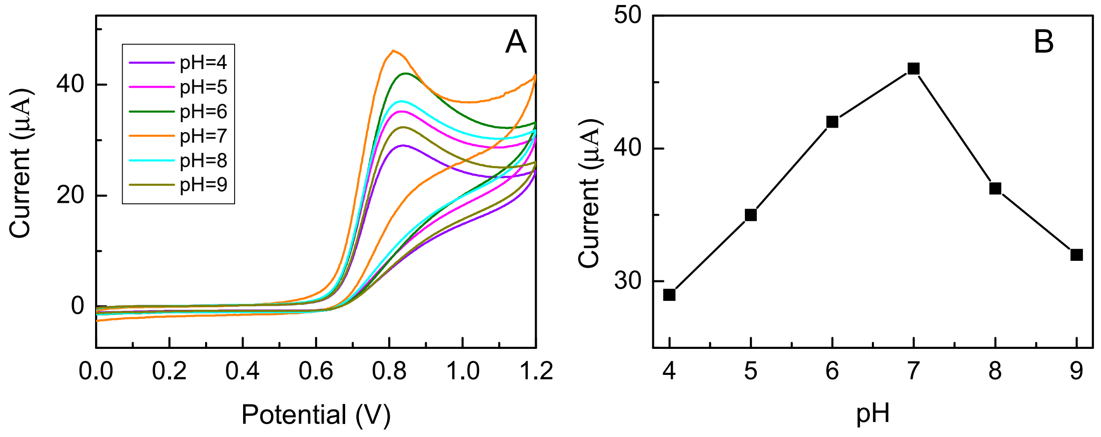

Figure. S5 (A) Cyclic voltammograms of Pd-ZnO/GCE in various pH solution containing 0.5 mM nitrite at scan rate of 50 mV/s, pH values from 4-9, respectively. (B) Effect of pH on the oxidation peak current of 0.5 mM nitrite at the Pd-ZnO/GCE.
